# Successful Treatment of Bronchial Dehiscence With Endobronchial Stent in Lung Transplantation

**DOI:** 10.1155/DTE.6.183

**Published:** 2000

**Authors:** G. M. Ferraroli, M. Ravini, M. Torre, L. Valvassori, P. A. Belloni

**Affiliations:** 1 Department of Thoracic Surgery Niguarda Ca' Granda Hospital Milan Italy; 2 Department of Neuroradiology Niguarda Ca' Granda Hospital Milan Italy; 3 A. De Gasperis Center Department of Thoracic Surgery Niguarda Ca' Granda Hospital P. zza Ospedale Maggiore Milan 3-20162 Italy

## Abstract

Bronchial dehiscence in lung transplantation is still a significant and threatening cause of morbidity, even if several progresses have been made in this field. In the present report we discuss a case of incomplete dehiscence of the right bronchial anastomosis in a patient who underwent sequential double lung transplantation for bronchiectasis. This complication has been successfully treated with endobronchial stent positioning, with the aim to allow the healing of the anastomosis around a rigid endobronchial support and to prevent the airway stenosis. The usefulness of 3D spiral CT reconstruction of bronchial tree is also underlined, for its capacity to detect the dehiscence and to monitor the healing of this complication.


*Diagnostic and Therapeutic Endoscopy*, Vol. 6, pp. 183–188
Reprints available directly from the publisher
Photocopying permitted by license only

© 2000 OPA (Overseas Publishers Association) N.V.
Published by license under
the Harwood Academic Publishers imprint,
part of The Gordon and Breach Publishing Group.
Printed in Malaysia.

### *Case Report*

### Successful Treatment of Bronchial Dehiscence with Endobronchial Stent in Lung Transplantation

G.M. FERRAROLI[a,*], M. RAVINI[a], M. TORRE[a], L. VALVASSORI[b] and P.A. BELLONI[a]

[a]*Department of Thoracic Surgery,* [b]*Department of Neuroradiology, Niguarda Ca' Granda Hospital – Milan, Italy*

*(Received 16 July 1999; Revised 26 October 1999; In final form 18 January 2000)*

**Bronchial dehiscence in lung transplantation is still a significant and threatening cause of morbidity, even if several progresses have been made in this field. In the present report we discuss a case of incomplete dehiscence of the right bronchial anastomosis in a patient who underwent sequential double lung transplantation for bronchiectasis. This complication has been successfully treated with endobronchial stent positioning, with the aim to allow the healing of the anastomosis around a rigid endobronchial support and to prevent the airway stenosis. The usefulness of 3D spiral CT reconstruction of bronchial tree is also underlined, for its capacity to detect the dehiscence and to monitor the healing of this complication.**

*Keywords:* Bronchial dehiscence, Bronchial stent, Lung transplantation, 3D spiral CT

### INTRODUCTION

Although improvements in patient selection, lung preservation, surgical technique, and immunosuppression have led to a decrease in the incidence of airway-related complications, bronchial dehiscence still remains a significant and threatening cause of morbidity in lung transplant recipients. The lung is the only solid organ that does not receive a direct vascularization during transplantation, and it was established that it needs almost 4 weeks to discover arterial circulation to the bronchial anastomosis, depending on retrograde collateral flow from the pulmonary circulation of the donor lung. Therefore, it is possible that in some circumstances the bronchial anastomosis could not adequately heal, leading to different complications (stenosis, malacia, granulation tissue, dehiscence). Among them dehiscence is certainly the most difficult to treat, especially when it is wide and clinically significant.

We report a case of incomplete discontinuity of the right bronchial anastomosis in a patient who

* Corresponding author. A. De Gasperis Center, Department of Thoracic Surgery, Niguarda Ca' Granda Hospital, P. zza Ospedale Maggiore, 3-20162 Milan, Italy. Tel./Fax: ++39-02-64442271.

underwent sequential double lung transplantation for bronchiectasis. The dehiscence, recognized by fiberoptic bronchoscopy, was successfully treated by stent positioning, with the aim to allow the healing of the anastomosis around a rigid endobronchial support and to prevent the airway stenosis. In the present case we also underline the usefulness of 3D spiral CT in addition to fiberoptic bronchoscopy in the detection of bronchial dehiscence and in the monitoring of its healing.

### CASE REPORT

The patient was a 36-year-old man who underwent sequential bilateral lung transplantation for bronchiectasis. The donor was a 48-year-old man who died of intracranial hemorrhage. Graft lung preservation was obtained by flushing with University of Wisconsin solution at 4°C, preceded by infusion of Prostaglandin E1 500 mg. Ischemic time was 128 min for the first graft and 254 min for the second. The surgical procedure was sequential bilateral lung transplantation according to the technique described by the Toronto Lung Transplantation Group [1]. Bronchial anastomosis was performed with absorbable 4/0 running suture in the membranous portion and absorbable 4/0 interrupted suture in the cartilaginous portion; the recipient's peribronchial tissue was wrapped around to reinforce the anastomosis. The operative and the early postoperative periods were uneventful. The recipient was extubated on the 2nd postoperative day and was discharged from ICU on the 4th postoperative day. Immunosuppression consisted of Cyclosporin, Azathioprine and antilymphocyte globulin. Methylprednisolone (500 mg IV) was administered at the beginning of surgical procedure and oral prednisone was started on the 5th postoperative day.

On the 20th postoperative day, an incomplete dehiscence of bronchial anastomosis was observed during surveillance bronchoscopy (Fig. 1). The patient was asymptomatic at that time and neither pneumothorax nor pneumomediastinum was

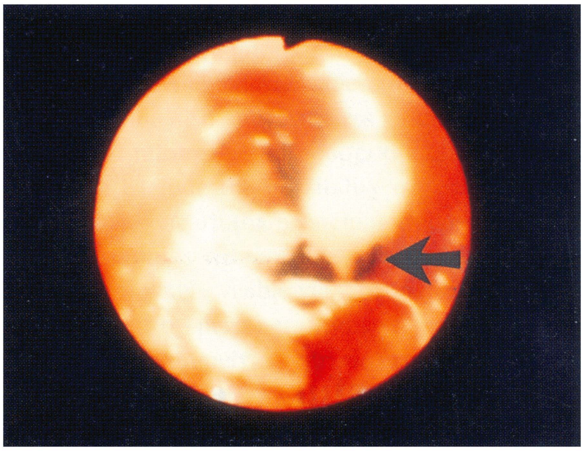

FIGURE 1 Right bronchial anastomotic dehiscence (arrow) originating from the middle of the membranous portion and involving the cartilaginous right portion for about 1 cm. Presence of fibrin that covered the anastomosis but not interfered with the air flow to the donor right bronchi.

present on chest X-ray. The discontinuity extended from the middle of the membranous bronchus toward the right, involving the cartilaginous bronchus for about 1 cm. The remaining portion of the anastomosis was adequately tight, covered by fibrin that partially reduced the lumen. Beyond the anastomosis, the donor's bronchus mucosa appeared well vascularizated.

On the basis of these data, in rigid bronchoscopy and under general anesthesia a silicone stent (Bronchial Stent with Posts, BSP-1240, 10 × 30 mm, Hood Laboratories, Pembroke – USA) was placed, bridging the airway breakdown (Fig. 2). The patient underwent chest CT which identified an air-filled cavity outside the bronchial lumen contained by the peribronchial tissue wrapping the anastomosis. A CT scan was obtained with an Elscint CT Twin Flash. Routine 10 mm scan intervals were obtained of the entire chest using the spiral technique. Additional volumetric 2.7 or 1.3 mm thin-section CT scans of the anastomosis were performed, with 50% overlapping to allow good visualization of the anatomic structures and pathologic changes in the reconstructed images. Volume-rendering 3D reconstructions were obtained off-line on a Silicon Graphics workstation.

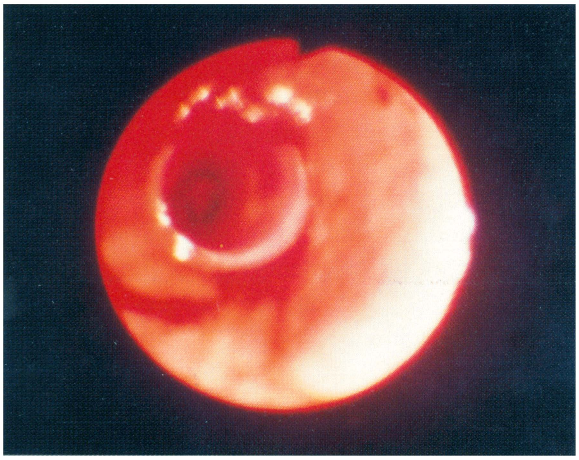

FIGURE 2 Bronchial stent (Dumon) placed at level of right bronchial anastomosis, where the dehiscence is present.

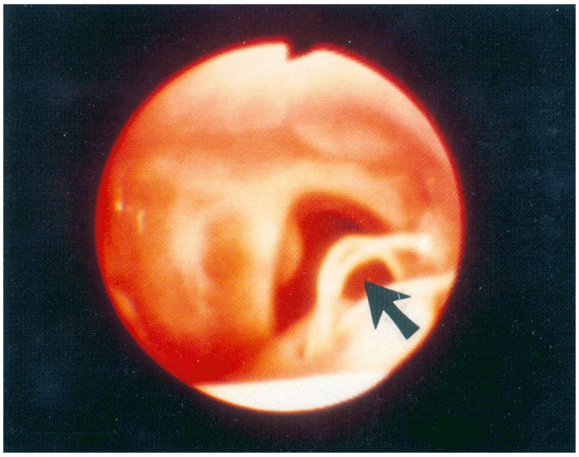

FIGURE 3 Bronchial anastomosis at 1 year: bronchial lumen with adequate caliber. Residual suture string (arrow) that does not interfere with bronchial patency.

The patient did not develop complications related to the airway disease. Bronchoscopic surveillance during the following weeks allowed us to observe the progressive reduction of the dehiscence whilst airway patency was maintained by the stent. In September 1998, 90 days after the starting event, the patient underwent another CT scan, which confirmed the healing of the defect and the resolution of the air collection outside the bronchus. A recent CT control in December 1998 showed no further change of the anastomosis, with a right bronchus perfectly patent and with no extraluminal collections. On the basis of these data, 6 months after transplantation the stent was removed.

At present (1 year after transplantation) the patient is in good health and the anastomosis, which has healed perfectly, keeps an adequate diameter (Fig. 3).

### DISCUSSION

Despite the improving results in lung transplantation, the healing of bronchial anastomosis still remains a problem. In fact, the probability of mucosal necrosis and anastomosis dehiscence is increased by prolonged ischemic time and interruption of bronchial vascularization. The reported incidence of bronchial complications ranges from 12% to 17% [2], in spite of the different solutions adopted to prevent them, such as direct revascularization of the bronchus [3], telescoping anastomosis, shortening of the bronchial stump, anastomosis wrapping with omentum, intercostal muscle, pericardiac fat, peribronchial tissue, etc. [4–7].

When the dehiscence is wide and associated with a severe clinical status, surgical repair is mandatory [8], whereas in cases of necrosis and partial dehiscence conservative treatment is generally preferred. In the latter case, apart from bronchoscopic monitoring, the laser and endobronchial stenting are frequently used [9], especially for the treatment of stenosis and malacia, which are the most frequent sequelae of dehiscence healing.

In the reported case the anastomotic dehiscence appeared to be less than 50% of the circumference. In the absence of signs and symptoms and owing to the good cicatrization of the remaining portion of the anastomosis, we decided to adopt a conservative treatment. A silicon endobronchial stent was placed just across the bronchial anastomosis, with the inferior end at level of the origin of the right upper bronchus. This procedure was justified by the well-known tendency of the bronchial anastomosis to heal evolving in stenosis. The stent acted as an internal splint on which the cicatrization tissue models, favoring the dehiscence margins to join and keeping an adequate bronchial diameter. After 6 months the bronchoscopic follow-up showed the complete healing of the anastomosis without any evidence of malacia or stenosis; the stent was therefore removed. The last bronchoscopic examination (at 1 year from transplantation) has confirmed a wide bronchial caliber.

The availability of the 3D spiral CT at our institution induced us to evaluate the usefulness of such a diagnostic tool in the study of the bronchial complication. In fact the 3D reconstructions and the possibility to obtain bronchogram-like images seem to apply very well in this field, in which very subtle defects in the anastomosis have a great impact in therapeutic decisions and the clinical outcome of the patient. 3D reconstructions "per se" do not obviously improve the diagnostic quality of the conventional axial images, but they can provide a better spatial visualization going beyond the limitations of the single transverse slices. The possibility of "cutting" the extraluminal tissue to firstly better understand the postoperative bronchial reorientation, secondly to evaluate bronchial wall alterations even after stent positioning, as well as recognition of mild changes in airway caliber, make this technique a complementary tool to both transaxial CT images and endoscopy [10,11] At the beginning of our case the images showed an air-filled cavity outside the bronchial lumen, contained by the peribronchial tissue wrapping the anastomosis (Fig. 4). After 6 months a new 3D spiral CT revealed the perfect healing of the dehiscence and the almost complete reabsorbtion of the extraluminal "cul de sac" (Fig. 5).

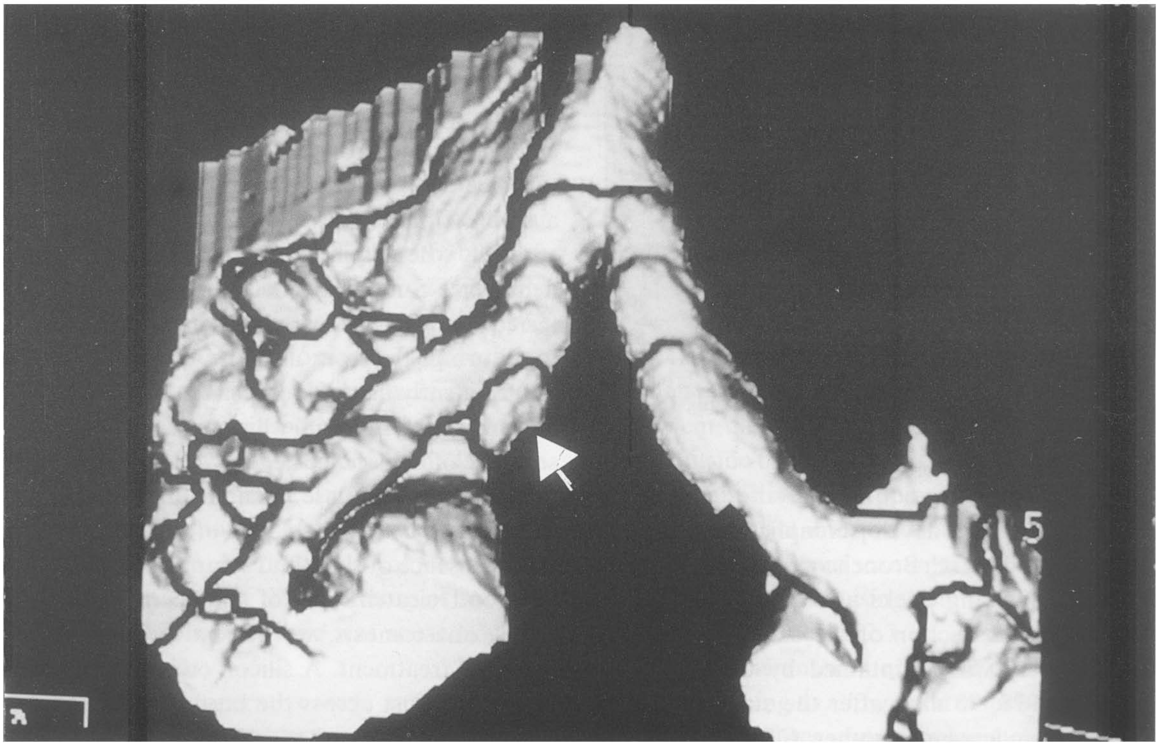

FIGURE 4 3D spiral CT reconstruction of bronchial anastomosis: presence of extrabronchial "cul de sac" at level of dehiscence (arrow), surrounded by peribronchial tissue.

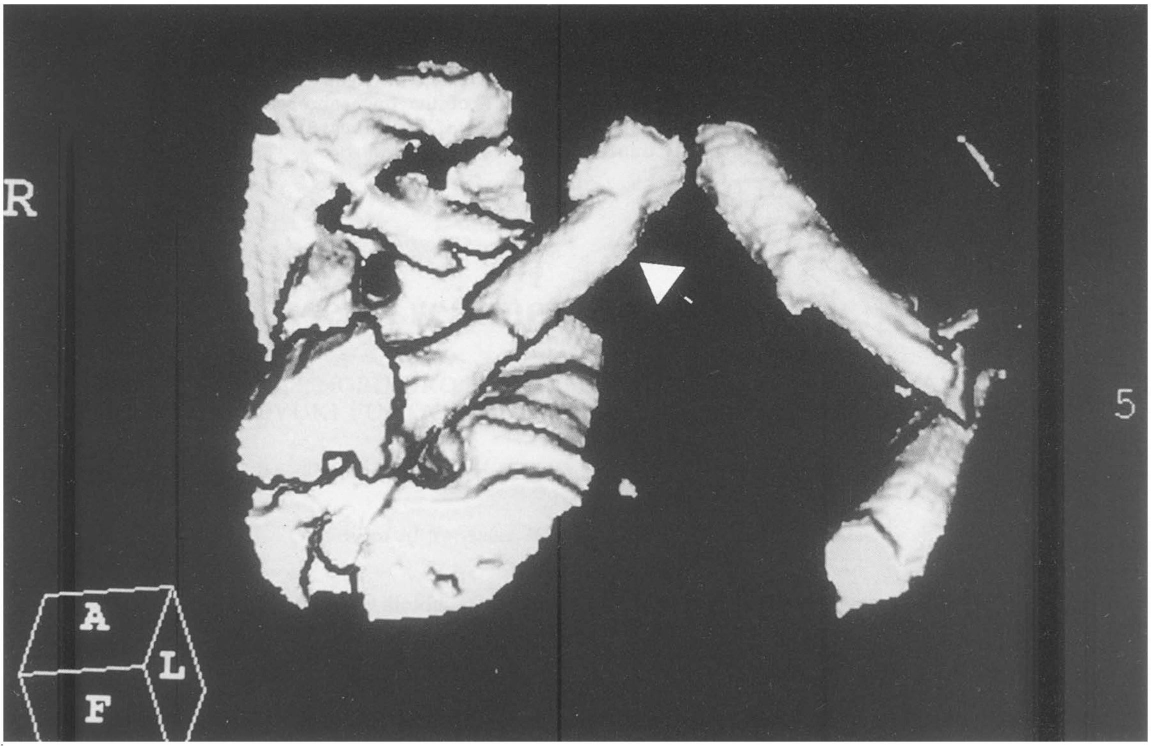

FIGURE 5 3D spiral CT reconstruction of bronchial anastomosis at 6 months that shows the disappearance of extrabronchial cavity (arrow) after treatment with endobronchial stenting.

### CONCLUSION

Partial dehiscence of bronchial anastomosis, if less than 50% of the circumference and not associated with signs or symptoms, may be effectively treated by endobronchial stent positioning, favoring the formation of cicatricial tissue around the internal splint, and preventing the formation of stenosis. This is a different option in the treatment of this kind of complication that could be considered, in very selected cases, an alternative method to the others.

Moreover, in our experience the 3D spiral CT has proved to be extremely useful both in the morphologic study of the dehiscence and in the evaluation of its healing.

### *References*

[1] The Toronto Lung Transplant Group: "Unilateral lung transplantation for pulmonary fibrosis". *N. Engl. J. Med.* 1986; **314**: 1140–5.

[2] Wilson, I.C., Hasan, A., Healey, M. *et al.* "Healing of the bronchus in pulmonary transplantation" *Eur. J. Cardiothorac. Surg.* 1996; **10**: 521–27.

[3] Daly, R.C. and McGregor, C.G.A. "Routine immediate direct bronchial artery revascularization for single-lung transplantation". *Annals of Thoracic Surgery* 1994; **57**: 1446–52.

[4] Pezzuoli, G. and Zannini, P. "Il trapianto di polmone" Ed. UTET, 1994; 139–57.

[5] Schmid, R.A., Boehler, A., Speich *et al.* "Bronchial anastomotic complications following lung transplantation: still a major cause of morbidity" *European Respiratory Journal* 1997; **10**: 2872–5.

[6] Kshettry, V.R., Kroshus, T.J., Hertz, M.I. *et al.* "Early and late airway complications after lung transplantation: incidence and management" *Annals of Thoracic Surgery* 1997; **63**: 1576–83.

[7] Patterson, G.A., Todd, T.R., Cooper, J.D. *et al.* "Airway complications after double lung transplantation" *J. Thorac. Cardiovasc. Surg.* 1990; **99**: 14–21.
[8] Shennib, H. and Massard, G. "Airway complications in lung transplantation" *Ann. Thorac. Surg.* 1994; **57**: 506–11.
[9] Colt, H.G., Janssen, J.P., Dumon, J.F. *et al.* "Endoscopic management of bronchial stenosis after double lung transplantation" *Chest* 1992; **102**(1): 10–16.
[10] Semenkovich, J.W., Glazer, H.S., Anderson, D.C. *et al.* "Bronchial dehiscence in lung transplantation: CT evaluation" *Radiology* 1995; **194**: 205–08.
[11] Lacrosse, M., Trigaux, J.P., Van Beers, B.E. *et al.* "3D spiral CT of the tracheobronchial tree" *Journal of Computer Assisted Tomography* 1995; **19**(3): 341–7.

